# Aging in a food-insecure country: why older adults in somalia are being left behind in the nutrition for healthy longevity agenda

**DOI:** 10.1016/j.jnha.2026.101000

**Published:** 2026-10-02

**Authors:** Maryan Ali Adam, Ayuub Axmed Osmaan, Mohamed Abdijabar Mohamed, Maryamo Maxamed Cali

**Affiliations:** a Faculty of Health Sciences and Tropical Medicine, Somali National University, Mogadishu, Somalia; b Benaadir Research, Consultancy & Evaluation Center, Mogadishu, Somalia

Dear Editor,

Healthy longevity has become a central global health objective, yet its nutrition agenda remains strikingly underdeveloped for older adults in fragile settings [1]. This omission is increasingly urgent because older age is tightly linked to frailty, disability, and multimorbidity, while food insecurity disrupts diet quality, meal regularity, and nutrient adequacy in ways that accelerate health decline [1].

Somalia exemplifies this neglected intersection. In Somalia, a nationally representative analysis of 52,154 participants found that 45.7% experienced food insecurity, with older adults showing elevated vulnerability and the pattern linked to the collapse of formal social security, displacement, and protracted instability [2]. Although urban residents fare better than rural and nomadic groups, urban residence is not protection against age-related nutritional exclusion when income insecurity, widowhood, limited mobility, and dependence on informal support converge in later life [2,3]. In sub-Saharan Africa, community studies indicate that 30–65% of older adults are malnourished or at risk, and frailty, sarcopenia, disability, and cognitive impairment are repeatedly linked to poor nutritional status and food insecurity [4]. Recent evidence published in The Journal of Nutrition, Health & Aging further shows that food insecurity is associated with increased odds of functional disability among older adults in Ghana and South Africa, highlighting the relevance of food access to functional health in later life [5].

This is not only a humanitarian concern; it is a longevity and health-systems issue. Global burden analyses show that sub-Saharan Africa bears a disproportionate burden of nutritional deficiencies in adults aged 65 years and older, with worse outcomes in lower-development settings [6]. Protein-energy malnutrition in older adults has increased globally since 1990 despite improvements in some micronutrient deficiencies, indicating that progress in nutrition has been uneven and insufficient for aging populations [6]. Recent work in The Journal of Nutrition, Health & Aging has also examined dietary protein intake and longitudinal frailty among home-dwelling older adults, reinforcing the importance of considering nutritional adequacy within healthy aging research [7]. Food insecurity in later life also appears to carry survival consequences: in a longitudinal cohort of adults aged 50 years and older, food insecurity was associated with a 41% higher risk of all-cause mortality and a 126% higher risk of premature mortality [3].

The central policy failure is conceptual. Current food-security responses in fragile settings still prioritize households, children, and emergency caloric relief, while older adults are rarely treated as a nutritionally distinct population with physiological vulnerability, functional limitations, and chronic disease risk [1,4]. Evidence from sub-Saharan Africa also remains geographically fragmented, with very limited intervention research and major underrepresentation of many settings, making invisibility part of the problem itself [4]. Recent evidence in The Journal of Nutrition, Health & Aging suggests that nutritional guidance may help prevent or improve frailty among older adults, particularly when interventions are individualized, professionally delivered, sustained over time, and combined with physical activity [8].

This issue deserves immediate scientific and policy attention because Somalia sits at the convergence of population aging, chronic food insecurity, displacement, socioeconomic vulnerability, and limited geriatric nutrition systems [1]. Ignoring older adults in this context will predictably increase frailty, disability, dependency, and avoidable mortality, while deepening inequities within humanitarian and health responses [3,4].

Priorities are clear: Somalia and its partners should integrate routine food insecurity and malnutrition screening for older adults into primary care, community outreach, and humanitarian services; build age-disaggregated surveillance; expand social protection and targeted food or cash support for widowed, isolated, and low-income older adults; and invest in context-specific nutrition-sensitive interventions, including dietary counseling, psychosocial support, and referral pathways for functional decline [2,3,8]. International healthy aging frameworks will remain incomplete until they explicitly include older adults in food-insecure settings such as Somalia.

Older adults in Somalia are not a peripheral population. They are a critical test of whether the healthy longevity agenda can reach people living at the intersection of fragility, poverty, food insecurity, and nutritional vulnerability.

## CRediT authorship contribution statement

**Maryan Ali Adam:** Conceptualization, Writing – original draft. **Mohamed Abdijabar Mohamed:** Conceptualization. **Maryamo Maxamed Cali:** Literature review. **Ayuub Axmed Osmaan:** Writing – review & editing. **All authors:** Final review and approval of the manuscript.

## Declaration of Generative AI and AI-assisted technologies in the writing process

During the preparation of this manuscript, the authors used generative AI and AI-assisted technologies solely to support language editing, grammatical refinement, and improvement of the clarity and organization of the manuscript. All AI-assisted content was critically reviewed and revised by the authors, who retain full responsibility for the accuracy, originality, interpretation, and integrity of the final manuscript. No generative AI or AI-assisted technology was used to generate, manipulate, or fabricate scientific data, results, analyses, or references. The authors remain fully responsible for the content of the manuscript.

## Declaration of competing interest

The authors declare that they have no competing interests that could have influenced the conduct, analysis, interpretation, or reporting of this work.
